# Spread of Multidrug-Resistant Bacteria by Moth Flies from Hospital Waste Water System

**DOI:** 10.3201/eid2608.190750

**Published:** 2020-08

**Authors:** Thomas Rupprecht, Annette Moter, Alexandra Wiessener, Joerg Reutershan, Klaus Lang-Schwarz, Michael Vieth, Christian Rupprecht, Ruediger Wagner, Thomas Bollinger

**Affiliations:** Klinikum Bayreuth, Bayreuth, Germany (T. Rupprecht, J. Reutershan, K. Lang-Schwarz, M. Vieth, T. Bollinger);; Friedrich-Alexander University, Erlangen, Germany (T. Rupprecht, M. Vieth);; Charité-Universitätsmedizin, Berlin, Germany (A. Moter, A. Wiessener);; Eberhard Karls University, Tübingen, Germany (J. Reutershan);; University of Oxford, Oxford, UK (C. Rupprecht);; Universität Kassel, Kassel, Germany (R. Wagner);; University Hospital Schleswig-Holstein, Lübeck, Germany (T. Bollinger)

**Keywords:** Antimicrobial resistance, antimicrobials, bacteria, *Clogmia albipunctata*, disease reservoir, infection control, moth flies, multidrug resistant bacteria, operating rooms, *Psychodidae alternata*, sewage, virulence factors

## Abstract

We documented and analyzed moth fly occurrence and spread of multidrug-resistant bacteria in a tertiary care hospital in Germany. The moth flies (*Clogmia albipunctata*) bred in the sewage system, then moved into the hospital, carrying biofilm and multidrug-resistant bacteria on their feet. Subsequently, the hospital developed a pest control protocol.

Hospital-acquired infections caused by multidrug-resistant (MDR) pathogens pose major challenges (*1*). Whereas the concept of transmission of pathogenic bacterial organisms through contact with medical staff is well established, other ways of spreading have not been sufficiently addressed. A tertiary care hospital in Germany observed sporadic outbreaks of MDR pathogens that could not be attributed to usual means of contamination. Concurrently, an increase in moth flies was observed. 

Psychodidae, the family that encompasses moth flies, includes a few species that can cause severe health problems, including the species *Psychoda alternata* and *Clogmia albipunctata*. Both species occur in large numbers where poor hygienic conditions exist, such as in sewage treatment plants, hospital waste water systems, or other environments where microbial biofilms exists, and therefore are considered nuisance pests (*2*). Reports suggest that wounds attract adult moth flies (*3*) and larvae have even been reported in samples from tear ducts (*3*). *C. albipunctata* moth flies have become a severe source of insect infestations in hospitals. One report (*4*) provides a summary of the permanent distribution of the species throughout Europe. 

Observations of both moth flies and outbreaks of MDR bacteria in the hospital were not initially linked. However, a newly constructed operating room (OR) could not be opened for use for ˃2 years due to occurrence of moth flies. The results of this study suggest that spreading of MDR bacteria by moth flies could explain these outbreaks. 

## The Study

We performed microbiologic analysis of all biofilm samples and moth flies by matrix-assisted laser desorption/ionization time-of-flight (MALDI-TOF) mass spectrometry (Bruker, https://www.bruker.com) and Phoenix automated microbiology system (Becton Dickinson, https://www.bd.com) according to European Committee on Antimicrobial Susceptibility Testing (EUCAST, https://eucast.org) guidelines. To visualize biofilms we used fluorescence in situ hybridization protocol on sections of embedded samples or moth flies (Appendix). We considered pathogens to be MDR if nonsusceptible to >1 agent in 3 of the defined categories, extensively-drug-resistant (XDR) if nonsusceptible to >1 agent in all but <2 of defined categories, and pan-drug resistant (PDR) if nonsusceptible to all listed antimicrobials (*5*). 

**Figure 1:**
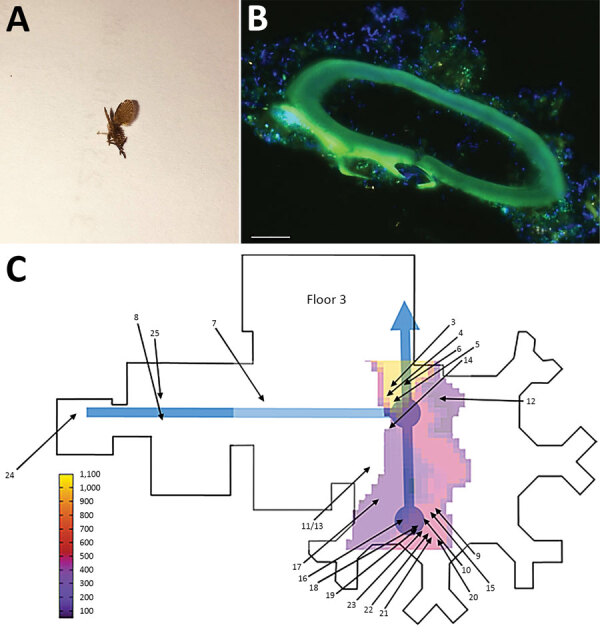
Investigation of multidrug-resistant bacteria spread by moth flies via biofilm in a hospital, Germany. A) Magnified *Clogmia albipunctata* moth fly. The length of the corpus is 2.5 mm. B) Fluorescence in situ hybridization (FISH) from biofilm of a sewage pipe with a blind end in the operating room (OR) using the pan-bacterial FISH-probe EUB338 labeled with fluorescein isothiocyanate (green), *Pseudomonas aeruginosa* specific probe labeled with Cy3 (orange), and nucleic acid stain DAPI (Thermo Fisher Scientific, https://www.thermofisher.com/) (blue). The oval structure seen is consistent with an eggshell of Psychodidae, which is colonized by bacteria. C) Schematic map of floor −3 (topographically representative of the hospital building). Blue lines represent the main sewage pipe; heat map shows the frequency of *C. albipunctata* occurrence merged from all floors of the building; blue arrow indicates sewage system discharge from the hospital; black arrows indicate where moth flies were captured on level –3. Arrow numbers correspond to the numbers in Tables 1, 2; arrows 3–6 indicate the position of the closed OR. The gradients in the heat map (summarized over all floors) point to the yellow region, which is 1 floor above the central sewage collection point of the hospital. The central sewage lines were inspected; we found biofilm and multiple moth flies at all investigated points.

We identified the moth flies as *C. albipunctata* (Figure 1, panel A) and found that they had entered the hospital room from a forgotten shunt between the drains and the waste air system (Appendix). To confirm that sewage pipes contained bacterial biofilms and moth fly eggs, samples were taken from the pipes, fixed, and stained. Psychodidae eggs were found in the biofilm (Figure 1, panel B). Infestation with moth flies seemed to increase with proximity to the central drain (Figure 1, panel C). Furthermore, we observed that moth flies were able to pass through the water-filled siphon of a bedpan washer (Video 1) and we identified extensive biofilm in the drains (Video 2). 

**Video 1:** Multiple moth flies occurring through a drain of a bedpan-washer at location 6 in Table 1. The drain was chemically cleaned before and the bedpan-washer had been closed the previous 3 weeks. Hence, entry of the moth flies from the OR can be excluded (adult moth flies are only viable a maximum of a few days).

**Video 2:** Extensive biofilm occurrence at location 4 in Table 1. Endoscopy taken using a disposable bronchoscope (Ambu aScope, https://www.ambu.com/).

**Figure 2:**
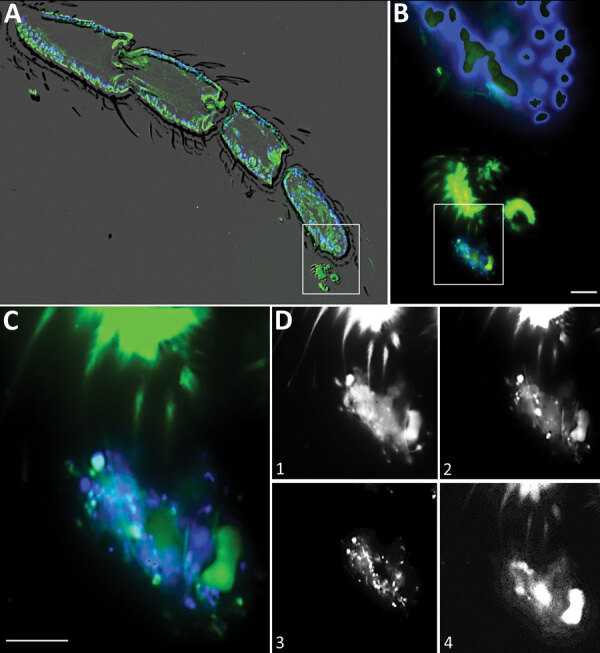
Fluorescence in situ hybridization (FISH) from a longitudinal section of a leg of a *Clogmia albipunctata* moth fly from a hospital in Germany. The fly was caught in the hospital, embedded, and stained (Appendix, https://wwwnc.cdc.gov/EID/article/26/8/19-0750-App1.pdf). A) Overview showing an overlay of the fluorescent images with a phase contrast to visualize the limbs of the legs. B) Higher magnification of the inset from panel A shows the anatomy of the tarsus and claws with an adjacent biofilm, which is stained by the bacterial probe (green) and DAPI (blue). C) Higher magnification of the inset from panel B shows the biofilm. Blue represents DAPI staining of DNA; bacteria were stained green with pan-bacterial FISH probe EUB338-FITC, *Enterobacterales* stained orange with an *Escherichia coli*–specific FISH probe (data not shown), and NONEUB (nonsense EUB) probe labeled with Cy5 was used to exclude unspecific probe binding. D) Overlay of the DAPI and fluorescein isothiocyanate channel shows the biofilm with different bacterial morphotypes. Different planes of the z-stack in the green channel (pan-bacterial probe) of the identical microscopic field depicts the different claws embracing the biofilm (D1 and D2). D3 shows the DAPI filter-set only with the DNA of the bacteria, whereas D4 shows the autofluorescence in the Cy5 filter-set NONEUB probe.

Fluorescence in situ hybridization and fluorescence microscopy on sections of embedded moth flies showed the presence of biofilms and bacteria on their feet (Figure 2). Not every moth fly caught was tested; however, representative moth flies from each identified location were tested. Furthermore, biofilm in sewage pipes revealed a kind of ecosystem consisting of moth fly larvae, vermicular, fungi, and bacteria (Appendix Figure 1). Subsequently we collected and microbiologically analyzed *C. albipunctata* and biofilm from different parts of the hospital (Tables 1, 2). Of the moth flies we analyzed microbiologically, we found that 41.1% carried MDR or even XDR bacteria (Appendix). Overall, 43.9% of specimens were MDR or XDR. 

**Table 1:** Occurrence of moth flies in a hospital, Germany*

No.	Psychodidae larvae/eggs	Count†	Type of room (comment)	Floor‡
1	Adult	1	Sewage line service opening (under OR 1)	−4
2	Larvae and eggs	>100	Sewage line service opening (200 m distant from OR 1)	−4
3	Adult	>50	OR 7§	−3
4	Adult	>500	Washroom, OR 7	−3
5	Adult	>50	Corridor, OR 7	−3
6	Adult	>50	Supply rooms for OR 7	−3
7	Adult	3	Corridor connecting ORs 1–6	−3
8	Adult	1	Corridor to ICU 58	−3
9	Adult	>20	Toilet (A3–40)	−3
10	Adult	>5	Doctor’s room (A3–43) St 35	−3
11	Adult	>10	Toilet (G3–58)	−3
12	Adult	>10	OR dermatology (B3–61)	−3
13	Adult	>500	Washing room G3–62	−3
14	Adult	>500	Toilet A3–06	−3
15	Adult	>5	Supply room (A3–41)	−3
16	Adult and eggs	>500 (>100 eggs)	Shower (F3–07) St 35	−3
17	Adult	>500	Shower floor 35 (F3–31), patient room and corridor	−3
18	Adult	>10	Bathroom floor 34 (E3–07)	−3
19	Adult	>10	Shower floor 34 (E3–08)	−3
20	Adult	>10	Clean supply room floor 34 (E3–01)	−3
21	Adult	>10	Kitchen floor 34 (E3–02)	−3
22	Adult	>10	Staff room floor 34 (E3–04)	−3
23	Adult	>10	Doctor‘s room floor 34 (E3–05)	−3
24	Adult	>5	ICU floor 58 W3001	−3
25	Adult	3	OR 12 heart surgery¶	−3
26	Adult	>500	Sluice to hospital kitchen (S2–20)	−2
27	Adult	>500	Hospital kitchen toilets (S2–20a, b)	−2
28	Adult	>10	Supply room emergency department (R1–52)	−1
29	Adult	>10	Kitchen emergency department (R1–57)	−1
30	Adult	>10	Toilet emergency department (R1–55)	−1
31	Adult	>10	Patient rooms emergency department	−1

Patient numbers correspond to the numbers in Table 2 and Figure 1, panel C. ICU, intensive care unit; OR, operating room; St, suite. †Count of Psychodidae larvae and adult flies during June 2016–October 2018. ‡Floor numbers are negative because the hospital is in part built on a hill so that Floor 0 is the most top level (Floor 0 had no moth fly observations). §OR 7 had been closed for years until we eliminated the source of moth flies. ¶In OR 12 only 1 moth fly was found even after an intense search. From all immediately reported occasions 1–2 moth flies were caught and analyzed microbiologically (see Table 2 for results).

**Table 2:** Multidrug resistant bacteria on moth flies and biofilm in a hospital, Germany*

No.	Specimens tested	Location found	Species identified†	Can produce biofilm?	Resistance level‡	Macroscopic/ histologic findings
1	Biofilm	Sewage line	*Achromobacter xylosoxidans*	Yes	Not classified	Biofilm, *Candida*, mucin, eggs
2	Biofilm	Sewage line	*Pseudomonas *spp.	Yes	Potential XDR§	Biofilm, cocoons, eggs
			*Escherichia coli*		MDR	
			*Lysinibacillus fusiformis*			
3	Moth fly	OR 7	*Bacillus *spp., *Citrobacter freundii*	Yes	MDR	NA
4	Biofilm	Endoscopy of sewage lines in washroom, OR 7	*Advenella species*	Yes	Potential XDR§	Extensive biofilm in all sewage lines, mucin, *Candida*
			*Bacillus *spp.	Yes		
			*Pseudomonas aeuginosa*	Yes		
			*Stenotrophomonas maltophilia*			
	Moth fly	Washroom OR 7	Sterile	NA	ND	NA
5	Moth fly	Corridor OR 7	*P. mosselii* *S. maltophilia*	Yes	Potential XDR§	NA
7	Moth fly	Corridor OR 1-6	*Bacillus *spp. *B. megatariume*	Yes Yes	ND	NA
8	Moth fly	Corridor ICU CS	*Bacillus *spp.	Yes		NA
9	Moth fly	Toilet (A3-40)	*S. maltophilia*		Potential XDR§	
	2 moth flies	Toilet (A3-40)	Sterile	NA	ND	NA
10	Moth fly	Doctors room (A3-43)	*Bacillus *spp. *P. nitroreducens*	Yes Yes	ND	NA
	Moth fly	Doctors room (A3-43)	*B. cereus* *B. thuringienses*	Yes Yes	ND	NA
14	Moth fly	Toilet (A3-06)	*Bacillus *spp. *B. cereus* *Chryseobacterium indologenses*	Yes	ND	NA
16	Biofilm	Shower drain	*B. cereus* *Bacillus *spp*.* *P. putida*	Yes	ND	Biofilm, eggs, *Candida albicans*
	Moth fly	Shower (F3-07)	*Bacillus cereus* *L. sphaericus* *Staphylococcus epidermidis*	Yes	ND	NA
	2 moth flies	Shower (F3-07)	*S. maltophilia* *B. thuringiensis*	Yes	Potential XDR	NA
17	Moth fly	Shower (F3-31)	*Enterococcus faecium (vanA)* *Bacillus *spp. *S. maltophilia*	Yes	Possible XDR¶ Potential XDR§	NA
	Moth fly	Shower (F3-31)	*E. faecium*	Yes	MDR	NA
24	Moth fly	ICU floor C	Sterile	NA	ND	NA

*Numbers at left are patient numbers, which correspond to the numbers in Table 1 and Figure 1, panel C. CS, cardiosurgery; C, cardiology; ICU, intensive care unit; MDR, multidrug resistant; OR, operating room; NA, not applicable; ND, not detected; XDR, extensively drug resistant. †Microbiology, histology, and fluorescence in situ hybridization findings in the investigated specimens. Note that not every moth fly from the Table 1 count column was analyzed.  ‡Resistance levels defined in Magiorakos et al. (*13*). §Potential XDR means that the international standards have not been applied to the bacterium *S. maltophilia*. Additional details are available in the Appendix. ¶Possible XDR means that it could be XDR but not all antimicrobial categories were tested. Additional details are available in the Appendix.

We subsequently examined pest control options. Our first approach, removing biofilm from accessible pipes in the sewage system, did not successfully reduce or eliminate moth flies. Our second approach, mechanically and chemically cleaning all sinks and proximal sewage lines, also did not prevent periodic reoccurrence of moth flies. Our third approach was more successful. We flushed all sinks in the OR at the same time with 60°C hot water for 15 min/wk, daily during summer, which suppressed *C. albipunctata* moth flies in the OR but not in the rest of the hospital. 

## Conclusions

Infection with and colonization by MDR bacteria is an increasing challenge in health care (*6*). Psychodidae (moth flies) are small, 1–4 mm in size, and have been regarded as unharmful vermin except in highly sterile areas. Therefore, they have often been overlooked or ignored and not considered a high-consequence problem. 

The results of our study suggest a change in this point of view is needed. If generalized to other hospitals, our findings indicate that *C. albipunctata* moth flies in hospitals, combined with MDR, XDR, or PDR (pandrug-resistant) bacteria in biofilms, pose an underestimated threat. The danger from this symbiotic system between moth flies and these bacteria results from moth fly eggs and larvae living in biofilm that is contaminated by a patient’s bacterial flora. Furthermore, biofilms can rapidly grow and spread over distances kilometers in length (*7*) and are almost impossible to eradicate. In the third and fourth stages of development, larvae living in the biofilm can begin to move, thus overcoming the water barriers in showers, bathtubs, toilets, and other washing units. At this point, adult moth flies can enter the hospital (Video 1) and transport drug-resistant bacteria from the microbial flora of the biofilm into the hospital. 

We frequently found *Stenotrophomonas maltophilia* on *C. albipunctata* moth flies and also in clinical samples from deep respiratory material, wounds, blood culture, urine, and bile. In 1 patient, for example, we found hospital-acquired *S. maltophilia* and a genetically identical strain in drains in a ward ≈250 m away (data not shown). Even though this evidence is scant, it does support our hypothesis. 

In addition, low doses of antimicrobials excreted by patients can result in the quick development and spread of plasmids (resistance genes) and virulence factors in biofilms (*8*). This process might result in resistance developing not only in a patient’s microbiota but also in hospital biofilm. Our observations suggest that the adult *C. albipunctata* moth flies can move freely throughout sewage systems and that they carry bacterial biofilm on their feet. Many authors have suggested the existence of missing links in polyclonal outbreaks and in other hard-to-explain observations (*9**,**10*). We hypothesize that moth flies in symbiotic combination with biofilms could, in part, explain one such observed transmission. However, the findings of this study are limited by the moderate number of moth flies, which should be addressed in future investigations.

Currently, there are no proven strategies, including chemical methods, to prevent or eradicate moth flies in sewage systems. However, weekly or, during summer, daily flushing with hot water (60°C) for 15 min was sufficient to suppress the moth flies in our study. We propose a prevention protocol including flushing weekly or daily with hot water (60°C), mechanical removal of biofilms; deconstruction of unused siphons or replacement by heatable siphons; and checking for unexpected outlets, such as drill holes, from drains into hospital rooms. These measures will not eliminate but might substantially suppress the problem. Once moth flies leave the drains, among the few available biofilms are patient wounds. Research has reported that adult moth flies are attracted to them, and *C. albipunctata* larvae have been found in wounds (*11**,**12*). Searching for moth flies and determining their microbial load might be advisable, especially if an unexpected bacterial outbreak occurs. Finally, our observations should be taken into account in the planning of hospital sewage systems in the future. 

Appendix Additional details about methods and results

## References

[1] Gerlich MG, Piegsa J, Schäfer C, Hübner NO, Wilke F, Reuter S, et al. Improving hospital hygiene to reduce the impact of multidrug-resistant organisms in health care—a prospective controlled multicenter study. BMC Infect Dis. 2015;15:441. 10.1186/s12879-015-1184-5 26493394 PMC4619269

[2] Faulde M, Spiesberger M. Hospital infestations by the moth fly, *Clogmia albipunctata* (Diptera: Psychodinae), in Germany. J Hosp Infect. 2012;81:134–6. 10.1016/j.jhin.2012.04.006 22560402

[3] El-Dib NA, El Wahab WMA, Hamdy DA, Ali MI. Case report of human urinary myiasis caused by *Clogmia albipunctata* (Diptera: Psychodidae) with morphological description of larva and pupa. J Arthropod Borne Dis. 2017;11:533–8. 29367929 PMC5775159

[4] Oboňa J, Balážovž L, Cáfal R, Dobránsky M, Filipovič P, Ivčič B, et al. Additions to the range expansion of the invasive moth midge *Clogmia albipunctata* (Williston, 1893) in Slovakia (Diptera Psychodidae). Folia faunistica Slovaca 17, 387–391.

[5] Magiorakos A-P, Srinivasan A, Carey RB, Carmeli Y, Falagas ME, Giske CG, et al. Multidrug-resistant, extensively drug-resistant and pandrug-resistant bacteria: an international expert proposal for interim standard definitions for acquired resistance. Clin Microbiol Infect. 2012;18:268–81. 10.1111/j.1469-0691.2011.03570.x 21793988

[6] Cassini A, Högberg LD, Plachouras D, Quattrocchi A, Hoxha A, Simonsen GS, et al.; Burden of AMR Collaborative Group. Attributable deaths and disability-adjusted life-years caused by infections with antibiotic-resistant bacteria in the EU and the European Economic Area in 2015: a population-level modelling analysis. Lancet Infect Dis. 2019;19:56–66. 10.1016/S1473-3099(18)30605-4 30409683 PMC6300481

[7] Vlamakis H, Chai Y, Beauregard P, Losick R, Kolter R. Sticking together: building a biofilm the *Bacillus subtilis* way. Nat Rev Microbiol. 2013;11:157–68. 10.1038/nrmicro2960 23353768 PMC3936787

[8] Madsen JS, Hylling O, Jacquiod S, Pécastaings S, Hansen LH, Riber L, et al. An intriguing relationship between the cyclic diguanylate signaling system and horizontal gene transfer. ISME J. 2018;12:2330–4. 10.1038/s41396-018-0183-0 29899518 PMC6092375

[9] Clarivet B, Grau D, Jumas-Bilak E, Jean-Pierre H, Pantel A, Parer S, et al. Persisting transmission of carbapenemase-producing Klebsiella pneumoniae due to an environmental reservoir in a university hospital, France, 2012 to 2014. Euro Surveill. 2016;21:30213. 10.2807/1560-7917.ES.2016.21.17.30213 27168586

[10] McBain AJ, Bartolo RG, Catrenich CE, Charbonneau D, Ledder RG, Rickard AH, et al. Microbial characterization of biofilms in domestic drains and the establishment of stable biofilm microcosms. Appl Environ Microbiol. 2003;69:177–85. 10.1128/AEM.69.1.177-185.2003 12513993 PMC152421

[11] Taylan-Ozkan A, Babur C, Kilic S, Nalbantoglu S, Dalkilic I, Mumcuoglu KY. Urogenital myiasis caused by *Psychoda albipennis* (Diptera: Nematocera) in Turkey. Int J Dermatol. 2004;43:904–5. 10.1111/j.1365-4632.2004.02051.x 15569013

[12] Tu W-C, Chen H-C, Chen K-M, Tang L-C, Lai S-C. Intestinal myiasis caused by larvae of *Telmatoscopus albipunctatus* in a Taiwanese man. J Clin Gastroenterol. 2007;41:400–2. 10.1097/01.mcg.0000212615.66713.ba 17413610

[13] Falagas ME, Koletsi PK, Bliziotis IA. The diversity of definitions of multidrug-resistant (MDR) and pandrug-resistant (PDR) *Acinetobacter baumannii* and *Pseudomonas aeruginosa.* J Med Microbiol. 2006;55:1619–29. 10.1099/jmm.0.46747-0 17108263

