# Supplementary figures and images for: Spread of Multidrug-Resistant Bacteria by Moth Flies from Hospital Waste Water System
Source: Emerg Infect Dis. 2020 Aug;26(8):1893–8. doi: 10.3201/eid2608.190750 (PMC7392454; doi:10.3201/eid2608.190750)

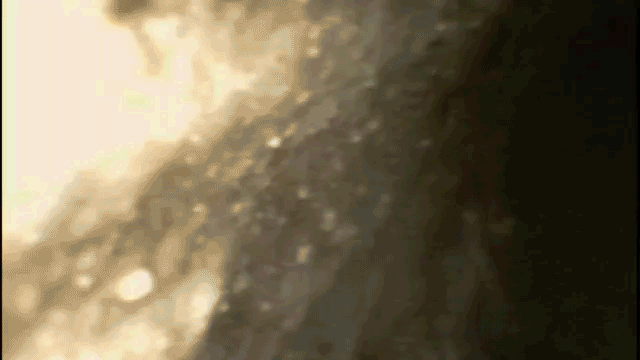

Supplement: Supplementary file 1 [file 19-0750-vid1.gif]

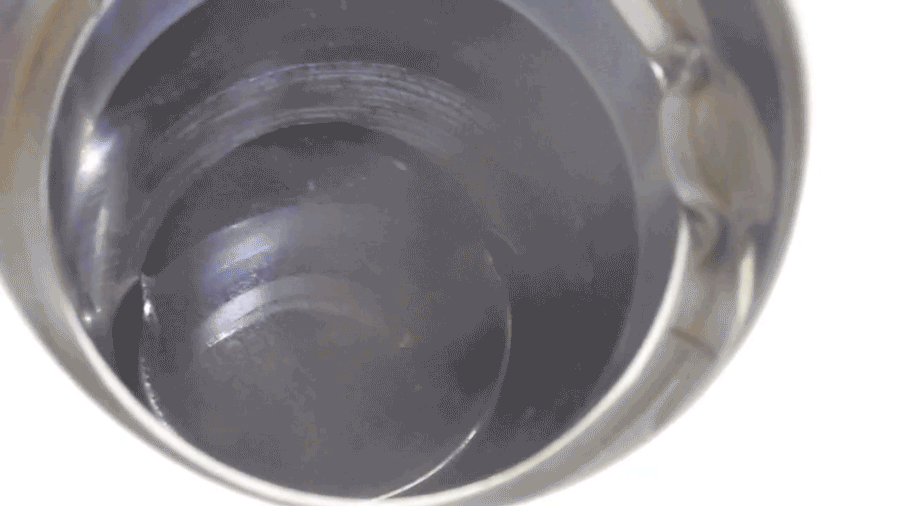

Supplement: Supplementary file 2 [file 19-0750-vid2.gif]
