# Supplementary material for: Spread of Multidrug-Resistant Bacteria by Moth Flies from Hospital Waste Water System
Source: Emerg Infect Dis. 2020 Aug;26(8):1893–8. doi: 10.3201/eid2608.190750 (PMC7392454; doi:10.3201/eid2608.190750)
Supplement: Appendix — Additional details about methods and results [file 19-0750-Techapp-s1.pdf]

Article DOI: https://doi.org/10.3201/eid2608.190750

Spread of Multidrug-Resistant Bacteria by Moth Flies from Hospital Waste Water System

Appendix

Material and Methods

Identification of moth fly sources

We completely isolated the operating room (OR) from the rest of the building by fly-proof enclosure. We closed and reopened all input and output connections (water pipes, air conditioning, drainage pipes, sewage lines) step by step to identify the source of moth flies. We also closed all sinks in the OR with tape. We did this to catch moth flies on the sticky site of the tape and thereby identify all sinks where moth flies might have left the siphons. The fluff filter of the waste air system was used as a sentinel for moth flies. Members of the task force did weekly documented inspections to identify the moth fly source and to examine the efficiency of pest control measures. In the rest of the hospital, the medical staff was advised to search for and to report the presence of moth flies. Finally a heat map was calculated (Gnuplot bilinear interpolation, http://www.gnuplotting.org/) to show the gradients of the count of fly occurrence and to locate the primary source.

Taxonomic identification of moth flies

The taxonomic identification of *Clogia albipunctata* was done by one of the author (R.W.) who is an expert on Psychodidae. For the identification moth flies were fixed in 80% ethanol and analyzed by microscope by 200x magnification. The size, wing shape, coloration, and genitalia were analyzed and the moth flies were identified by the expert knowledge of R.W. as *C. albipunctata* (*1*). For further illustration we depict a life cycle of *C. albipunctata* in Appendix Figure 2.

Collection of moth flies and biofilm samples and analysis

Moth flies were collected and characterized histopathologically and microbiologically using classical culture techniques and fluorescence in vitro hybridization (FISH) analysis. Furthermore, the central occurrence sites of the sewage lines were inspected and sampled

distally to the water barriers (siphons) by endoscopy (Ambu aScope, https://www.ambu.com/).

Microbiologic Culture Techniques

All specimens were investigated by classical microbiologic cultures. Hence, moth flies were captured in sterile tubes with 25 mL PP container yellow screw caps (Sarstedt, https://www.sarstedt.com/), flooded with 8 mL brain-heart-infusion bouillon (BD, https://www.bd.com/), and then incubated at 35°C for 20 h. Subsequently, 50 μL of brain-heart-infusion was plated on selective agar for VRE-Agar (MAST Diagnostica, https://mast-group.com/), MRSA-Agar (MAST Diagnostica), ESBL-Agar (bioMerieux, https://www.biomerieux.com/), and TSA (BD) subsequently plates were incubated for additional 20 h. Bacterial species were identified by MALDI-TOF (Bruker, https://www.bruker.com/), antimicrobial resistance was tested applying the Phoenix-System (BD) and result interpretation was done according to EUCAST clinical breakpoints (www.EUCAST.org). Antimicrobial resistance genotype for VRE was determined applying *a vanA*/B-PCR (Cepheid, https://www.cepheid.com/).

Pest control measures

To find appropriate and cost-effective pest control measures we took advantage of the closed OR which was a well-controlled setting. In addition, only the task force members were authorized to access the operation room and this was controlled by sealing all rooms after each inspection (weekly). The untouched seals were photo documented at the beginning of the next inspection.

Our first approach to pest control, mechanically removing biofilm from accessible pipes in the sewage system, did not successfully reduce or eliminate moth flies. Our second approach, mechanically and chemically cleaning all sinks and proximal sewage lines with pyrethroid insecticide, also did not prevent periodic reoccurrence of moth flies. Our third approach was more successful. We flushed all sinks in the OR at the same time with 60°C hot water for 15 min/wk (daily during summer) which suppressed *C. albipunctata* in the OR but not in the rest of the hospital.

Definitions of Multidrug-Resistant-Organism

We used the definitions in Magiorakos et al. (*2*), which were developed from a consensus of international experts from the US Centers for Disease Control and Prevention and the European Center for Disease Prevention and Control. Definitions were set only for

the most common bacteria and include definitions for multidrug-resistant (MDR), extensively drug-resistant (XDR) and pandrug-resistant (PDR) bacteria. In brief, we considered pathogens to be MDR if nonsusceptible to ≥1 agent in 3 of the defined categories, extensively-drug-resistant (XDR) if nonsusceptible to ≥1 agent in all but ≤2 of defined categories, and pan-drug resistant (PDR) if nonsusceptible to all listed antimicrobials. "Possible" is used to modify MDR, XDR or PDR means that the drug resistance definition could be met, but not all listed antimicrobials have been tested.

However, neither *Stenotrophomonas maltophilia* nor *Pseudomonas* spp., which we found in sewage lines as well as on moth flies, are included in this definition. Because we tested according to EUCAST rules and for *S. maltophilia* EUCAST provides only breakpoints for co-trimoxazole, it is difficult to classify this bacterium. To address this problem, we also list the breakpoints for all tested antimicrobials which demonstrates that, except for co-trimoxazole, none is susceptible when interpreted by Clinical & Laboratory Standards Institute criteria. In addition, *S. maltophilia* is MDR even though only in part these resistances are acquired. To address this problem we classified the *S. maltophilia* isolates as "potential" XDR, acknowledging that this is more a proposal than a fact. The same descriptor (potential XDR) was applied to *Pseudomonas* spp. as the international classification is defining criteria only for *P. aeruginosa*.

Fluorescence in vitro hybridization

Biofilm samples and moth flies were fixed in FISH-fixation solution (MoKi Analytics GmbH, Berlin, Germany) at 4°C for 3–6 days, embedded in cold polymerizing methacrylate resin and sectioned in 2µm sections as described (*3*). Sections were first screened with the pan-bacterial, 16S rRNA directed probe EUB338 (*4*), which detects most bacteria and EUK516 for detection of Eukarya (*5*). To exclude unspecific probe binding, positive FISH signals were reviewed using the NONEUB (nonsense EUB) probe NON338 (*3*). For genus- or species-specific detection of bacteria we used probes specific for *P. aeruginosa* (PSMG) (*6*), *Enterobacterales* (EC1531) (*7*), and *Staphylococcus* (STAPHY) (*6,8*). The nucleic acid stain DAPI (4',6-diamidino-2-phenylindole) was applied as a counterstain to visualize microorganisms and eukaryotic cell nuclei.

Supplemental results

Moth fly source in the OP

We identified 2 possible ways of moth fly invasion which were the air conditioning and the drains. However, biofilm, which is essential for moth flies, was only found in the sewage lines. Most likely there was a shunt between the sewage system and the waste air system due to a removed autoclave. After the shunt was closed, we detected no further moth flies in the OR (operation room). Furthermore, we noticed that the prolonged shut down of the OR facilitated moth flies to leave the drains through sinks into the OR (Video 1, https://wwwnc.cdc.gov/EID/article/26/8/19-0750-V1.htm). Closing sinks with tape proved that moth flies escaped the sewage pipes at all sinks in the OR because several moth flies were caught on the sticky side of the tape. It was concerning that moth flies can leave the drain, where MDR and XDR bacteria frequently occur. For this reason, we analyzed moth flies and sewage pipes for MDR, XDR, and PDR pathogens.

Moth fly identification

The moth flies were classified as male and female *C. albipunctata* (Figure 1, panel A).

References

1. Nilsson A. Aquatic insects of north Europe, vol. 1: Ephemeroptera, Plecoptera, Heteroptera, Megaloptera, Neuroptera, Coleoptera, Trichoptera and Lepidoptera. Stenstrup, Denmark: Apollo Books; 1996.

2. Magiorakos A-P, Srinivasan A, Carey RB, Carmeli Y, Falagas ME, Giske CG, et al. Multidrug-resistant, extensively drug-resistant and pandrug-resistant bacteria: an international expert proposal for interim standard definitions for acquired resistance. Clin Microbiol Infect. 2012;18:268–81. PubMed https://doi.org/10.1111/j.1469-0691.2011.03570.x

3. Wallner G, Amann R, Beisker W. Optimizing fluorescent in situ hybridization with rRNA-targeted oligonucleotide probes for flow cytometric identification of microorganisms. Cytometry. 1993;14:136–43. PubMed https://doi.org/10.1002/cyto.990140205

4. Moter A, Leist G, Rudolph R, Schrank K, Choi BK, Wagner M, et al. Fluorescence in situ hybridization shows spatial distribution of as yet uncultured treponemes in biopsies from digital dermatitis lesions. Microbiology. 1998;144:2459–67. PubMed https://doi.org/10.1099/00221287-144-9-2459

5. Mallmann C, Siemoneit S, Schmiedel D, Petrich A, Gescher DM, Halle E, et al. Fluorescence in situ hybridization to improve the diagnosis of endocarditis: a pilot study. Clin Microbiol Infect. 2010;16:767–73. PubMed https://doi.org/10.1111/j.1469-0691.2009.02936.x

6. Braun-Howland EB, Vescio PA, Nierzwicki-Bauer SA. Use of a simplified cell blot technique and 16S rRNA-directed probes for identification of common environmental isolates. Appl Environ Microbiol. 1993;59:3219–24. PubMed https://doi.org/10.1128/AEM.59.10.3219-3224.1993

7. Poulsen LK, Licht TR, Rang C, Krogfelt KA, Molin S. Physiological state of *Escherichia coli* BJ4 growing in the large intestines of streptomycin-treated mice. J Bacteriol. 1995;177:5840–5. PubMed https://doi.org/10.1128/JB.177.20.5840-5845.1995

8. Trebesius K, Leitritz L, Adler K, Schubert S, Autenrieth IB, Heesemann J. Culture independent and rapid identification of bacterial pathogens in necrotising fasciitis and streptococcal toxic shock syndrome by fluorescence in situ hybridisation. Med Microbiol Immunol. 2000;188:169–75. PubMed https://doi.org/10.1007/s004300000035

9. McAlpine JF, Peterson BV, Shewell GE, Teskey HJ, Vockeroth JR, Wood DM. Manual of nearctic Diptera, vol. 1. Ottawa, Ontario, Canada: Research Branch Agriculture Canada; Monograph 27; 1981.

Appendix Table. Resistance phenotypes of bacteria*

Bacteria	*Stenotrophomonas maltophilia*		*Pseudomonas* spp.		*Escherichia coli*		*Citrobacter freundii*		VRE	
Antimicrobials	MIC	C	MIC	C	MIC	C	MIC	C	MIC	C
Penicillin G	†	‡	†	‡	†	‡	†	‡	>0.25	R‡
Flucloxacillin	†	‡	†	‡	†	‡	†	‡	>2	R‡
Erythromycin	†	‡	†	‡	†	‡	†	‡	>4	R‡
Clindamycin	†	‡	†	‡	†	‡	†	‡	>1	R‡
Vancomycin	†	‡	†	‡	†	‡	†	‡	>8	R§
Teicoplanin	†	‡	†	‡	†	‡	†	‡	>8	R§
Linezolid	†	‡	†	‡	†	‡	†	‡	1	S¶
Daptomycin	†	‡	†	‡	†	‡	†	‡	2	R§
Quinopristin/ Dalfopristin	†	‡	†	‡	†	‡	†	‡	1	S¶
Ampicillin	>8	R‡	>8	R‡	>8	R§	>8	R§	>16	R§
Ampicillin/ Clavulanic acid	>32/2	R‡	>32/2	R‡	†	R‡	†	R§	†	R§
Ampicillin/ Sulbactam	†	R‡	†	†	†	R‡	†	R§	†	R§
Tircarcillin/ Clavulanic acid	†	‡	>64/2	R§	32/2	R§	>64/2	R§	†	R§
Piperacillin	>64	R‡	8	I‡	8	S‡	>64	R‡	>64	R§
Piperacillin/ Tazobactam	>64/4	R§	>8/4	I§	8/4	S‡	8/4	S¶	16/4	R§
Cefuroxime	>8	R‡	>8	R‡	>8	R§	†	R§	†	R‡
Ceftriaxone	>4	R‡	>4	R‡	>4	R§	>4	R§	†	R‡
Ceftazidime	16	R§	4	I§	16	R§	>16	R§	†	R‡
Cefepime	†	§	16	R§	16	R§	<1	S	†	R‡
Meropenem	>8	R§	0.5	S§	<0.125	S¶	0.25	S¶	†	R‡
Imipenem	>8	R§	>8	R§	<0.25	S¶	1	S¶	>8	R‡
Aztreonam	†	‡	>16	R§	>16	R§	>16	R§	†	‡
Ciprofloxacin	>1	R§	>1	R§	<0.25	S¶	>1	R§	>1	R§
Levofloxacin	>2	R§	>2	R§	≤0.5	S¶	>2	R§	1	R§
Cotrimoxazol	2/38	S¶	>4/76	R§	<1/19	S¶	<1/19	S¶	†	R‡
Gentamicin	4	R§	>4	R§	<1	S¶	<1	S¶	>4	R§
Tobramycin	4	R§	>4	R§	<1	S¶	<1	S¶	4	R§
Amikacin	8	R§	8	S§	<4	S¶	<4	S¶	†	R§
Fosfomycin	>128	R§	>128	R§	<16	S¶	<16	S¶	>64	R‡
No. AMR groups	6		7		6		6		6	
No. remaining AMS groups	1		0		5		5		2	
Antimicrobial groups not tested	2		1		5		5		3	
Interpretation	Potential XDR*#		Potential XDR*#		MDR#		MDR#		Possible XDR#	

*Term "potential XDR" introduced to classify multidrug–resistant *S. maltophilia* and *Pseudomonas* spp., which were not addressed in the international classification but do show extensive resistance phenotype and would fulfill the criteria of an XDR according to the definition for *Pseudomonas aeruginosa.* AMR, antimicrobial resistant; AMS, antimicrobial sensitive; C, category; I, increased exposure; MDR, multidrug resistant; MIC, minimum inhibitory concentration; R, resistant; S, sensitive; VRE, vancomycin-resistant *Enterococci*; XDR, extensively drug resistant

†No breakpoint for this antibiotic for the bacterium because of insufficient evidence or does not work for gram-negative bacteria

‡Not part of the classification for this bacterium

§Antimicrobial group has ≥1 AMR substance

¶Antimicrobial group AMS

#MDR determined according to EUCAST clinical breakpoints (version 10.0); XDR according to EUCAST classification and our extension

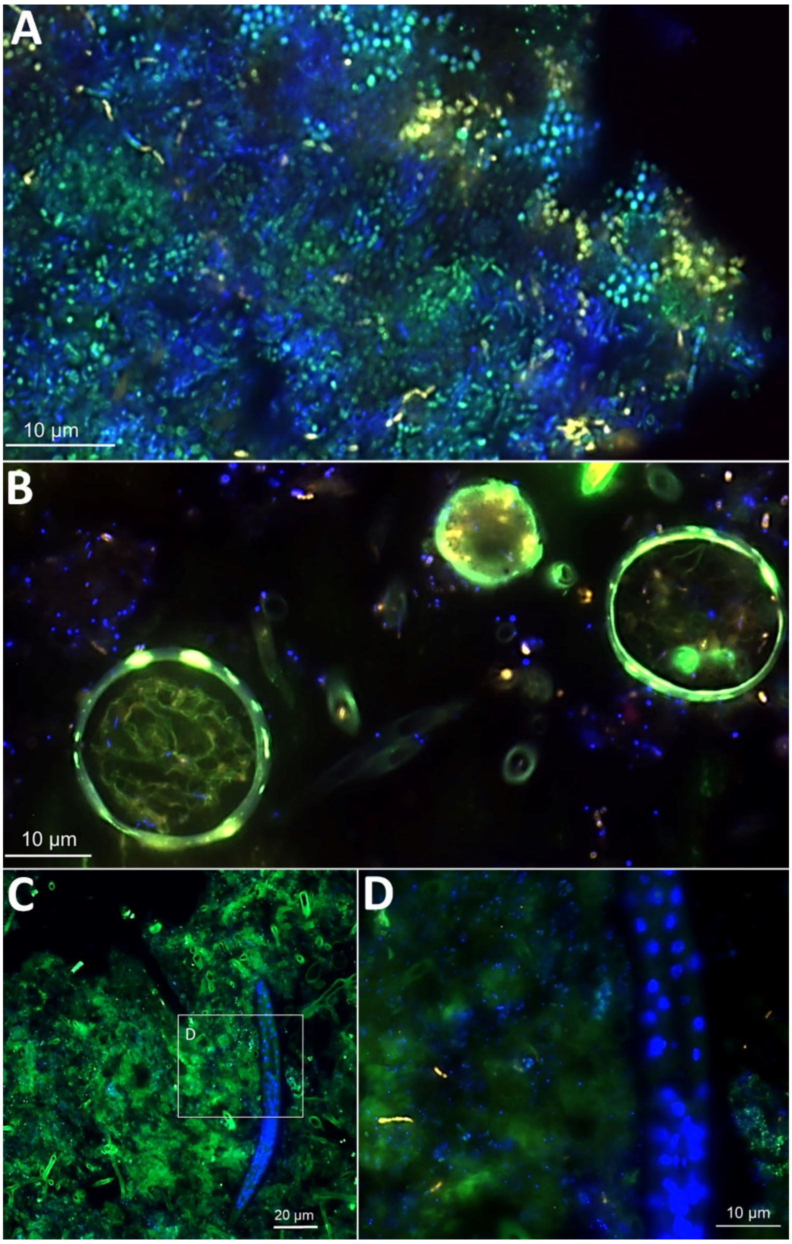

Appendix Figure 1. Fluorescence in situ hybridization (FISH) from a blind sewage pipe in the operation theater (No. 2 in Table 2) using the pan-bacterial FISH-probe EUB338 labeled with FITC (B, green), *Pseudomonas aeruginosa*–specific probe labeled with Cy3 (orange), and nucleic acid stain DAPI. (A) shows the rich biofilm with different morphotypes and microcolonies, including numerous *P. aeruginosa* cells scattered throughout the biofilm. (B) Biofilm sample taken from the sewage pipes (No. 16 in Table 2, shower sink,) with several eggs (not from *Clogmia albipunctata*). DNA is stained blue by DAPI, EUB338-Cy3 (orange) as pan-bacterial staining, Eukara were stained green (EUK516-FITC), negative binding control NON338-Cy5 (magenta, data not shown). (C) FISH image showing a mature biofilm sample taken from the sewage pipes (No.4 in Table 2, endoscopy of sewage lines, Table 2) with hyphae and a worm, most likely a nematode. With the green filter set, auto-fluorescent hyphae are visible in the biofilm material in the overview. The nematode is distinguishable by the nucleic acid stain DAPI. (D) At higher magnification *P. aeruginosa* is visible, detected by a specific Cy3-labeled FISH-probe (orange).

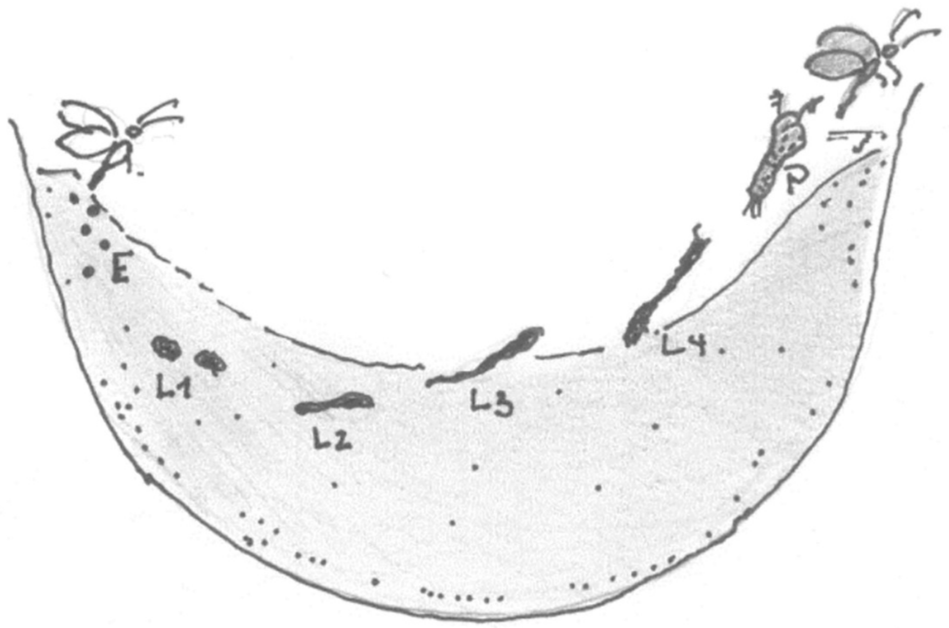

Appendix Figure 2. Life cycle of *Clogmia albipunctata*. The adult female moth fly lays up to 300 eggs (E). Larvae develop in 4 stages (L1–L4); during L3 and L4 they can move in the biofilm and water, fed by organic substances in the film. Moth fly pupae (P), the last larval stage, migrate to the water surface from where the new moth fly leaves the cocoon. The whole cycle lasts ≈3–4 weeks, depending on the environmental temperature (*1,9*).
